# Agro–ecosystem sensitivity to climate change over the Ethiopian highlands in a watershed of Lake Tana sub–basin

**DOI:** 10.1016/j.heliyon.2021.e07454

**Published:** 2021-07-02

**Authors:** Mintesinot Azene Taye

**Affiliations:** Bahir Dar University, P. O. Box: 5501, Bahir Dar, Ethiopia

**Keywords:** Climate, Environment, Agriculture, Livelihood, Upper Blue Nile basin

## Abstract

The study analyzed the level of agro–ecosystem sensitivity to climate change among the agro–climatic zones (ACZs) that are situated in the highlands of Lake Tana sub–basin. The analyses considered the actual land capability class (LCC in % considering soil texture, slope and elevation zone), crop diversity (count), length of growing period (LGP, month), and inter–annual variability of climate (mean annual rainfall–MARF in mm, mean monthly minimum temperature–MMMinT in ºC, and mean monthly maximum temperature–MMMaxT in ºC). For comparison purpose, it was essential to index/standardize the values of specified indicators. The proportion of arable land varied from 13.30% (in the Sub-Alpine) to 93.00% (in the Moist–Cold). The value of coefficient of variation showed the presence of variations of 7.85–11.21 (%), 7.21–10.34 (%), 16.37–39.61 (%) for MARF (mm), MMMaxT (ºC), and MMMinT (ºC), respectively across the ACZs. The inter–annual variability of both onset and offset time of rainy season was found to be in the range of 0.3–1.25 months. The LGP (month) was in the range of 3.25–6.25 across the ACZs; whereas crop diversity (count) ranged from 2–7. The production of red onion (allium cepa), oat (*Avena sativa*), local wheat (*Triticum*), and pea (*Pisum sativum*) was abandoned in the Sub–Alpine; whereas the production of linseed (*Linmu usitatisimum*), barley (*Hordeum vulgare*), and niger *(Guizotia abyssinica)* in the Moist–Cool. Yet, crops like maize and tef became the common crops in the Cold, possibly because of global warming. The indexed value of agro–ecosystem sensitivity to climate change ranged from 0.14–0.71. The level of agro–ecosystem sensitivity was higher towards the Sub–Alpine. The local development interventions to be made in the various ACZs need to be determined/prioritized considering the level of agro–ecosystem sensitivity.

## 1 Introduction

Ethiopia has reached a population of ~100 million with a growth rate of 2.5% [1, 2]. Agriculture contributes nearly 43% of the GDP, 80% of the employment, and 75% of the export in the country [3]. On top of that nearly 80% of the population in Ethiopia resides in the highland parts of the country [1]. In the meantime, more than 95% of the agricultural area in the Ethiopian highlands depends on almost exclusively on rainfall [4]. Frequent occurrence of extreme events such as drought and flooding and ecosystem degradation including soil and bio–diversity loss has been occurring [5, 6, 7] for reasons related to the increment of concentration of greenhouse gases (CO^2^, CH^4^ and N_2_O) in the atmosphere. The global average atmospheric and oceanic temperatures have increased by 1.1 °C above the pre–industrial period [8, 9]. Climate change mainly affects precipitation, temperature, evapotranspiration, and ultimately the whole hydrologic cycle and the capability of an agro–ecosystem [5, 10]. In the context of the current study, agro–ecosystem refers to the bio–physical environment (topography, climate, soil/land, crop diversity) that provide basic ecosystem services including food production to the rural community situated in a given agro–climatic zone of a watershed.

Change in climate and its adverse impacts are predicted to continue in East Africa [12], aggravating the existing challenges to satisfy the food demands of an ever–increasing population [13]. Climate change is evident in Ethiopia mainly through dramatic increment in surface temperature [14, 15, 16] that exacerbates recurrence of droughts [16, 17] and change in precipitation patterns [18, 19, 20, 21, 22]. The impact of environmental change is expected to be more visible in the Ethiopian highlands where the ecosystem is climatologically sensitive [10, 11, 12, 13].

Losses from disasters have reached one trillion US dollars at a global level since 2000 [23]. Yet, nearly 95% of humanitarian finance is still spent in the form of relief rather than a planned agro–ecosystem management [24]. The existence of climate variability in Northwestern part of Ethiopia was prevalent [11, 18, 25]. The mean maximum and minimum temperature of Ethiopia will increase by 2–2.3 and 0.8–0.9 °C in 2030 and 2.2–2.7 and 1.4–1.7 °C in 2050, respectively [26]. High levels of rainfall variability and drought is experienced locally and regionally in the headwater regions of the Nile in Ethiopia [11, 18, 27, 28] and internationally through its effects on downstream countries such as Sudan and Egypt [29]. Ethiopia has been facing climate–induced drought and stress on the productivity of crop and livestock, contributing to widespread food insecurity [30]. For instance, due to climate change related reasons, the total water yield of the basin is estimated to decrease by −1.7 to −6.5% [31]. The recent inter–annual climate variability (drought) in the years of 1965, 1972–73, 1983–84, 1987–88 and 1997 showed decline in agricultural production with serious degradation of the environment in the country [27].

The variability of climate, the properties of soil, terrain, and land management condition do have adverse effects on the capability of an agro–ecosystem [32, 33, 34, 35]. Socioeconomic drivers of land use change such as technological development, population growth and increasing per capita demand are projected to continue in the future. The expansion of misuse of land, large appropriation of multiple ecosystem services and the degradation of land and biodiversity are believed to be unprecedented in human history [36, 37, 38, 39]. All these climate change related events would adversely increase the degree of agro–ecosystem sensitivity to environmental change predominantly in rainfall dependent economies [16, 35]. Though the country's gross domestic product (GDP) is estimated to rise by 10% per year from 2010 to 2025 with the highest export growth driven by crop and livestock production [3], crop yields are still very low [28]. The amount of farmland per person is falling rapidly, as population growth places more pressure on a limited landscape. Agricultural progress is not keeping pace with population growth [2, 3]. Yet, the agricultural land use and ecosystem conservation practice lacks scientific procedures and legal mechanisms [41, 42, 43, 44]. As per the works of [45, 46], the unprecedented expansion of human settlement induced land use land cover (LULC) changes in the Ethiopian Highlands resulted in the total loss of habitat quality and habitat distribution”.

The land capability (the degree of land limitation) is governed by the different land attributes such as the types of soil, topography, climate etc. These attributes limit the extents of land available for various purposes [32, 33, 34]. Sustainable agriculture would be achieved if lands are categorized and utilized based up on their different use [33, 37, 41, 42]. Therefore, LCC provides a convenient checklist of the natural resource limitations that need to be considered when natural resource planning is undertaken in a given watershed [32, 34, 37, 45].

As agro–ecosystem entities are interdependent [32, 42], employing an agro–climatic zonation in environmental studies is a commendable scientific way–out [32, 36, 45, 47]. Watershed and agro–climatic zone (ACZ) based delineation of a study site would be desirable for integration of biophysical ecosystem [34, 36, 37, 49]. This scientific way–out is consistent with the recommendation of a resilient theory that intend to provide a path towards greater sustainability by embracing humans–in–nature perspective, uncertainty, variability, community fluctuation in response to disturbance and recognition of incomplete knowledge [50, 51]. As per the survey works of previous studies, the importance of agro–ecological based intensification rural development [47] and understanding possible environmental changes [48] for reducing the degree of vulnerability to climate change were justified. The use of agroecology in rural development strengthens the household adaptive capacity during adversities [47].

Yet, the previous studies missed to compare the level of agro–ecosystem sensitivity to climate change among ACZs of the Upper Blue Nile basin in a comprehensive manner. That is, for instance, land capability class (LCC), inter–annual climate variability (rainfall and temperature), length of growing period (LGP), and crop diversity were not taken into account. The objective of the current study was to analyze the level of agro–ecosystem sensitivity to climate change among the ACZs of the Gilgel Abay watershed taking into account the specified indicators. The output of this study would be an essential component to enhance the process of adaptation to climate change.

The current study is believed to be relevant to the contemporary science (international scientific community) for the fact that the study considers: 1) the most common and persistent problem (climate change and variability) that could be raised as a global forefront agenda; 2) a resilience theory that represents humans–in–nature perspective that guide change in social–ecological systems by adaptively design management strategies and policies; 3) the need to integrate various biophysical variables; 4) agro–climatic and watershed as geographic unit of analysis for a comparative study; and 5) possible local variability (among agro–climatic zones of a watershed) in the face of global climate change.

## 2 Methods and materials of the study

### 2.1 The Gilgel Abay watershed

**Figure 1:**
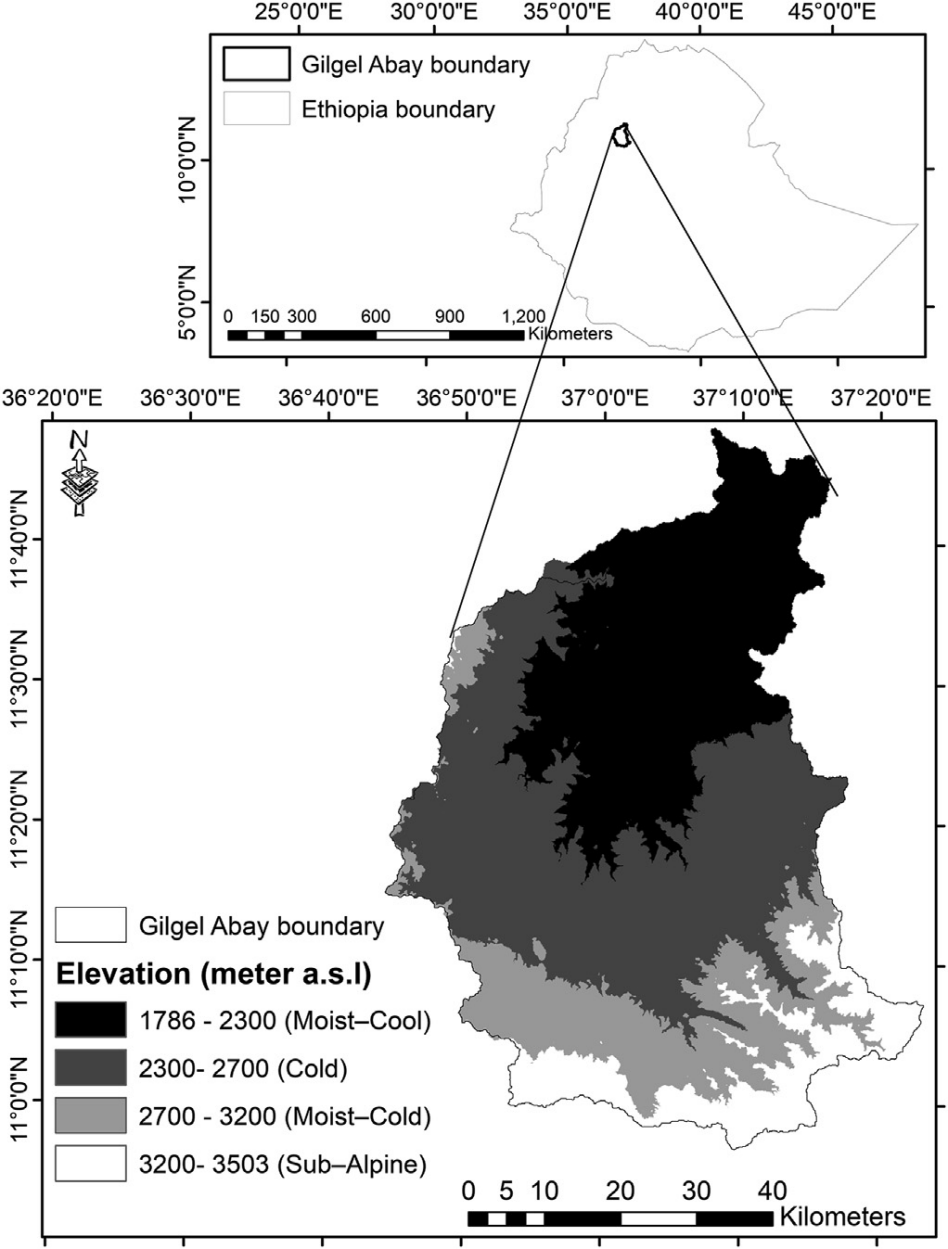
Location map of the agro–climatic zones of Gilgel Abay watershed, Ethiopia.

The Gilgel Abay watershed is situated in the Northwest highlands of Ethiopia, within 10.95°–12.78°N, and 36.89°–38.25°E (Figure 1). The watershed covers an area of 5,004 km^2^, and encompasses Moist–Cool (1786–2300 m a.s.l), Cold (2300–2700 m a.s.l), Moist–Cold (2700–3200 m a.s.l) and Sub–Alpine (3200–3503 m a.s.l) ACZs. The indicated information was also stated in previous studies [36, 50, 51].

Gentle slope, hilly landscape, and steep slope are the major landforms in the site. Cultivated land, shrubland, grassland, wetland, built–up area, forest and plantation land, and waterbody are the land use land covers of the site [51]. Cultivated land, shrubland, and grassland are the predominant ones. The mean annual total precipitation in the study site is ~1400 mm; whereas its MMMaxT and MMMinT are ~25 °C and ~7 °C, respectively. Nearly 80% of the total rainfall precipitates in June, July and August. The maximum temperature was recorded in March [52]. Alisols, Nitisols and Vertisols are the major soil types of the watershed [53, 54]. The majority of the population in the Gilgel Abay watershed depends on small scale food crop production; in the meantime, the population density in the site approaches to 189.4 persons/km^2^ [1].

### 2.2 Agro–ecosystem sensitivity to climate change

Different from the traditional thinking of ecological resistance and linear successional dynamics, a resilience theory represents humans–in–nature perspective that guide change in social–ecological systems by adapting design of management strategies and policies. Resistance describes the capacity of systems to remain unchanged by disturbance, while resilience is the capacity to return to a former configuration following a disturbance. Resilience recognizes the existence of threshold conditions that contribute to the formation of alternative stable states to minimize the degree of livelihood vulnerability to a given hazard [50, 51, 55, 56].

**Figure 2:**
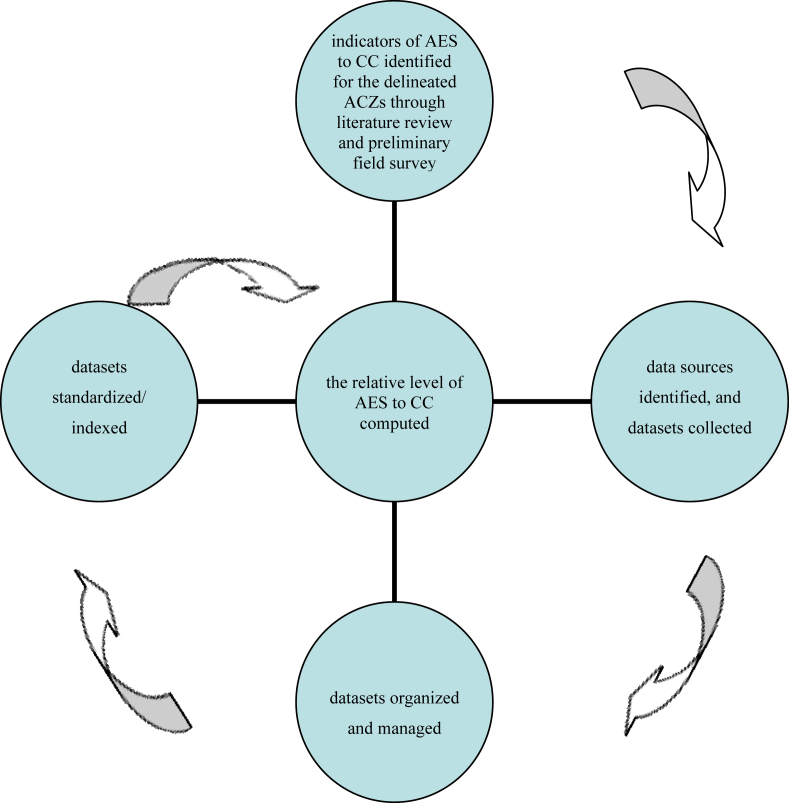
A flow chart of methodologies employed in the study. AES: agro–ecosystem sensitivity, CC: climate change, ACZs: agro–climatic zones.

Similar with previous works [37, 55, 56, 57], the current study assumes that livelihood vulnerability to climate change is a result of agro–ecosystem sensitivity to climate change/hazard, exposure to climate change and adaptive capacity. Yet, its scope is limited to analyzing the relative level of livelihood vulnerability to climate change in terms of agroecosystem sensitivity to climate change. Alike with what is stated in the work of [3, 37, 58], for the sake of the current study, sensitivity refers to the degree to which a system is affected, either adversely or beneficially, to climate–related stimuli. It is common to consider one factor like climate change in the comparative analysis of exposure, sensitivity and/or vulnerability. That is, being sensitive to climate change may not necessarily mean sensitive to any other environmental problem like change in vegetation cover/type or any other factor. The variables to be considered in the agro–ecosystem sensitivity could vary depending on the context of the study site and the available dataset. The actual LCC (slope–%, soil texture class, elevation zone–meter above sea level (m a.s.l)), and variability of climate in terms of inter–annual variability of mean annual rainfall (MARF, mm), mean monthly minimum temperature (MMMinT, °C) and mean monthly maximum temperature (MMMaxT, °C) were considered in the current study. Furthermore, the onset time of rainy season (being late by month), the offset time of rainy season (being earlier by month); the LGP (month), and crop diversity (count) were taken into account. Based on the preliminary field observation, and works of the previous studies [1, 3, 4], it was found essential to consider the mentioned indicators for the fact that the livelihood of people who live in the study site heavily depends on subsistence–based rain–feed agriculture. A flow chart for methodologies employed in the current study is presented (Figure 2).

#### 2.2.1 Land capability class (LCC)

**Figure 3:**
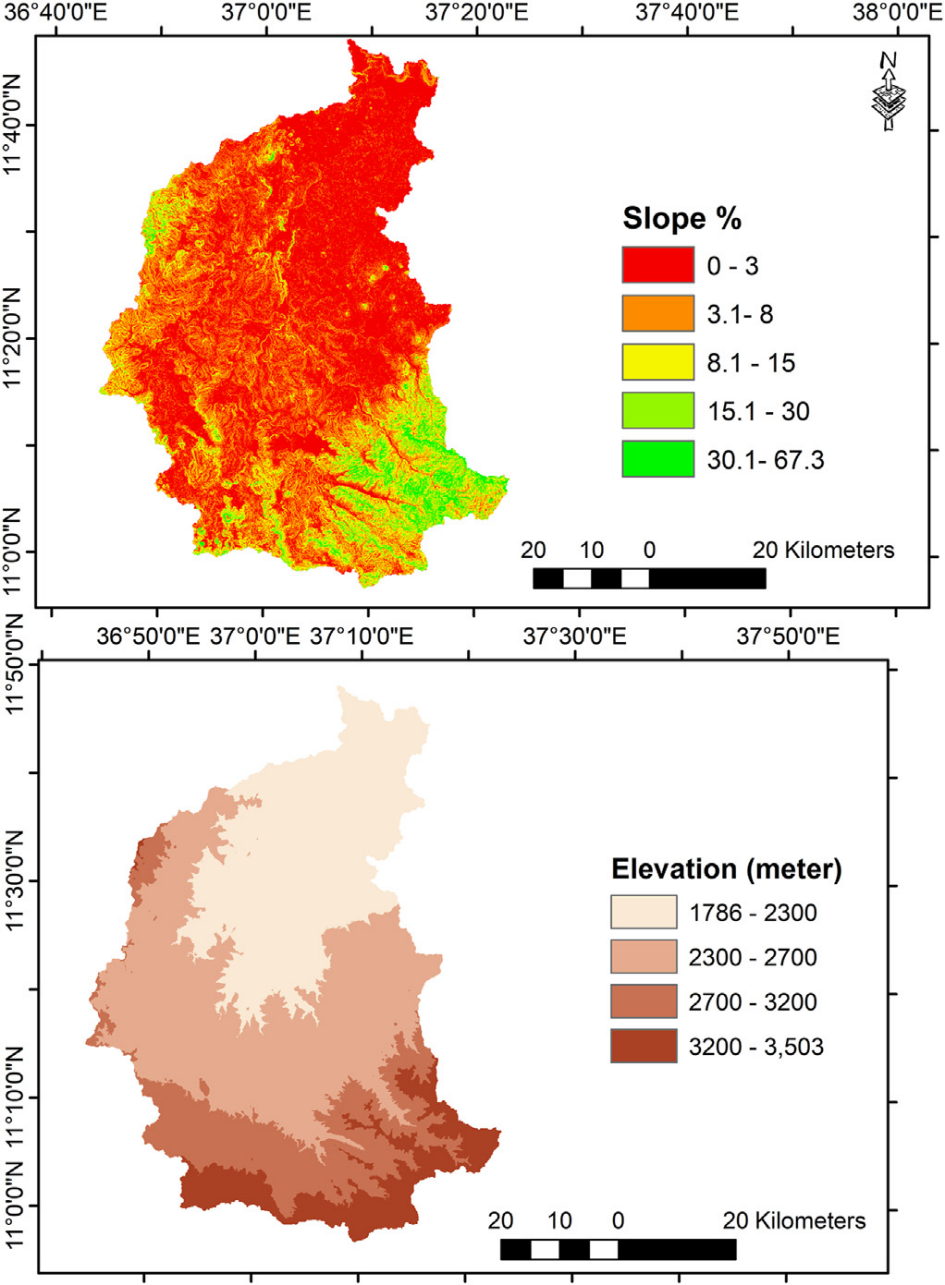
Slope (%) and elevation (meter a.s.l) classes considered for collecting sample soil in the Gilgel Abay watershed, Ethiopia.

**Figure 4:**
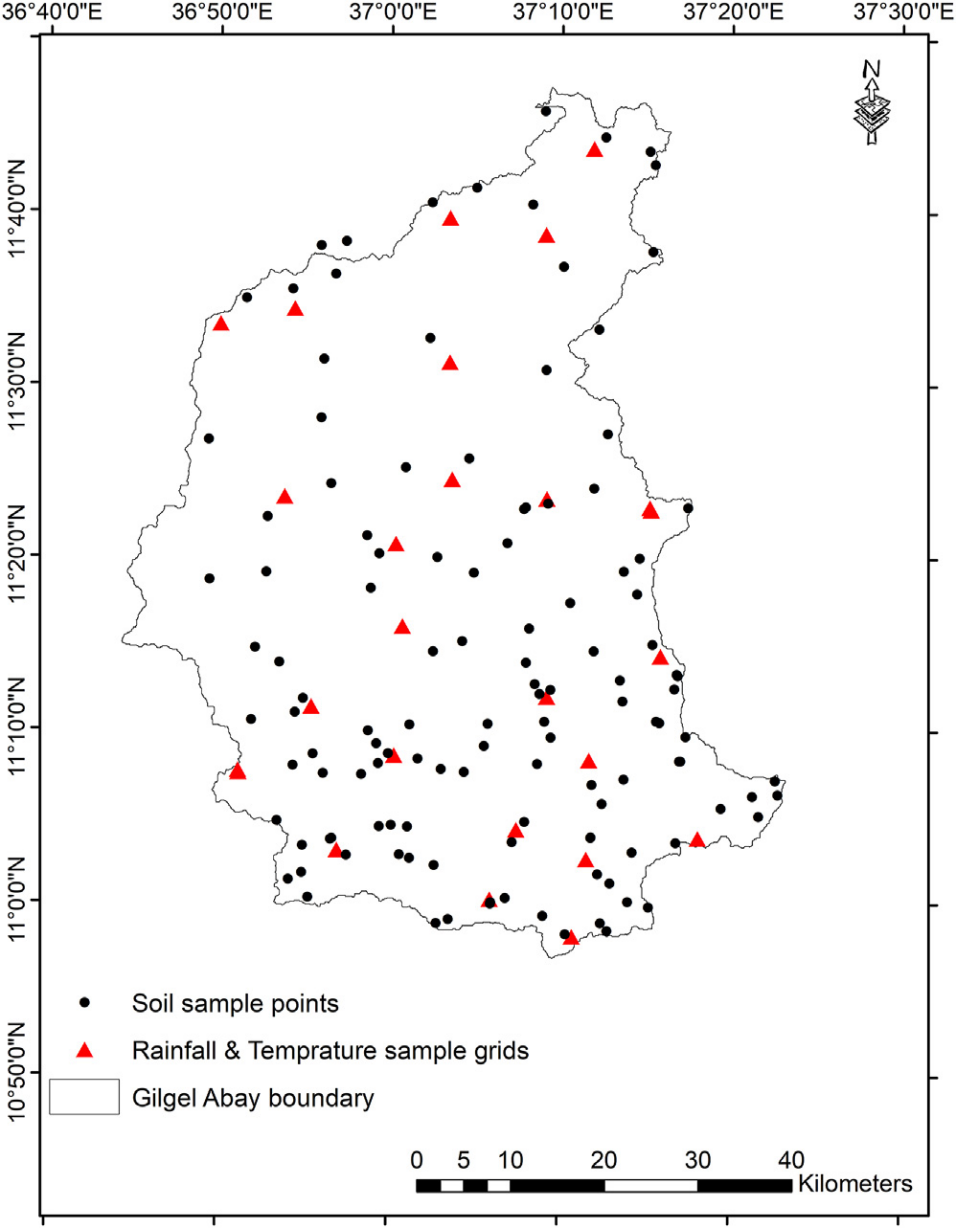
Sample points/grids considered for collecting soil and climate (rainfall and temperature) datasets in the Gilgel Abay watershed, Ethiopia.

In countries like Ethiopia where the economy directly and highly depends on rainfall agriculture, a sustainable land use is mandatory to enhance the capability of an agro–ecosystem [28, 37]. In this regard, the importance of considering a spatial variation in land resources for rural development was explained. Land is a source of water and soil resources that serves as a base for establishing a resilient livelihood system [59]. Land capability studies could be done considering a certain limitation of land depending on the context of the study site [40, 60, 61]. In the current study, LCC analysis was conducted taking into account classes of soil texture (%), slope (%) and elevation class (m a.s.l). Shuttle Radar Topographic Mission Digital Elevation Model (SRTM DEM–30m) was the source of satellite imagery for generating elevation and slope zones of the watershed. The sample units were decided taking into account slope classes in each of the ACZs. Accordingly, a total of 124 samples were collected with the help of hang auger from the stratified 6 slope classes across the ACZs (Figure 3 and Figure 4). To make ready the soil samples for laboratory analysis, first the texture samples were dried in an oven; then after the samples were sieved through a 2 mm sieve adopting the guidelines of ISRIC [62].

Ultimately, the measured soil texture class was decided by following the guideline of the USDA's soil texture triangle. After generating values for soil texture, slope and elevation classes, the LCC was determined. To combine layers of soil texture, slope zone and elevation classes for generating one LCC layer, equal weight overlay technique was used because of the absence of well–established reference to be used for assigning distinct weight to the identified components. Yet, the weight attached to the diverse indicators identified under each category was different (Table 1). The level of limitation and fitting LCC was assigned adapting the guideline of USDA [40, 63].

**Table 1:** Variables considered to determine the degree of land limitation for various LCCs in the Gilgel Abay watershed, Ethiopia.

Slope (%) and Soil Texture (%) classes	The assigned degree of limitation for various LCC (0–1)					
	I (0)	II (0.2)	III (0.4)	IV (0.6)	V (0.8)	VI (1)
Flat or almost flat (0–3)	x					
Gently sloping (3–8)		x				
Slopping/Moderately steep (8–15)			x			
Steep (15–30)				x		
Very steep (30–44.25)					x	
Clay loam		x				
Clay			x			
Heavy clay				x		

LCC: land capability class.

#### 2.2.2 Climate variability

Like what was indicated in the study of [36, 37, 57], the current study assumed the presence of negative association between the degree of climate variability and the performance of ecosystem services in general and amount of crop yield to be collected, in particular. The variability of climate in the Gilgel Abay watershed was analyzed taking into account the inter–annual variability of MARF (mm), MMMinT (°C) and MMMaxT (°C). Coefficient of variation (CV %) was employed to compare the level of climate variability among the ACZs of the study watershed. Alike with the works of [26, 36], the current study assumed the presence of negative association between the degree of climate variability and the performance of ecosystem services in general and amount crop yield to be collected in particular. The rainfall dataset was collected from Climate Hazards Group InfraRed Precipitation with Station grided data (CHIRPS v2), having a spatial resolution of ~0.05° (~5km∗5km) and a temporal coverage of 1981–2020. The temperature dataset was collected from Ethiopian Meteorological Station (EMA) with station grided data, having a spatial resolution of ~0.05° (~5km∗5km) and a temporal coverage of 1983–2018. The datasets of CHIRPS and EMA were used after referring their appropriateness from the previous study [18] and the report of Ethiopian meteorological agency, respectively. As to compute the average value of rainfall and temperature of each ACZ, 26 sample grid/station values were considered across the four ACZs of the watershed (Figure 3 and Figure 4). Data about the LGP (month), onset and offset time of rainfall (month) were also collected from the local elderly farmers using focus group discussion (FGD).

#### 2.2.3 Crop diversity and length of crop growing period

For purposes like backing the measured data with ground observation, considering the experience of local people is believed to be helpful in the study of local agro–ecosystem [64]. As stated in the resilience theory, local knowledge could be taken as empirical knowledge acquired over long–term experimentation, and is found to be a means to adapt local environmental changes [33].

In the current study, the actual crop diversity of major local food crops (in count) and their LGP (month) were considered for investigating the level of agro–ecosystem sensitivity to climate change among the ACZs of the Gilgel Abay watershed. Similar with the works of previous studies of [37, 65], the current study assumed the existence of direct association between crop diversity (count) and the performance of ecosystem services in absorbing unexpected environmental shocks. In identifying the major crops in various agroecological zones of the study watershed, the Ethiopian Central Statistics Authority (CSA) was referred. The validity of this data set was checked with information generated from the local farmers employing FGD. One FGD discussion involving of eight elderly local farmers was made in each ACZ of the study watershed.

Ultimately, in order to compare the relative level of agro–ecosystem sensitivity to climate change among the ACZs of Gilgel Abay watershed, it was essential to index and aggregate the values of indicators (Table 2). As presented by the previous works [55, 56, 66, 67], if various indicators of a vulnerability component are measured at different scales, it becomes essential to standardize the measured values of all indicators. The importance of standardizing values is to convert the indicators to relative rather than absolute values. Various indicators could have a direct or indirect direction of association with agro–ecosystem sensitivity to climate change (Table 2)". Various indicators could have a direct or indirect direction of association with agro–ecosystem sensitivity to climate change (Table 2). For instance, longer LGP will make an agro–ecosystem more sensitive to climate change, and it would have a direct association. On the other hand, more crop diversity will make an agro–ecosystem less sensitive to climate change, and it would have an indirect association. In such cases, it essential to invert the values of indicators that will have an indirect association (Eq. (1)).

(1) Crude Inverse Value=(1/(Observed Value+1))

**Table 2:** Indicators of agro–ecosystem sensitivity to climate change and their expected associations.

Indicators of agro–ecosystem sensitivity to climate change	Expected direction of association with agro–ecosystem sensitivity to climate change
LGP (month)	Direct: the longer the LGP of a given crop in a given place, the higher the possibility of being exposed to climate variability. Hence, the agroecosystem of a place with shorter LGP will be less sensitive to climate change. That is, the crop to be grown will get matured and harvested within a short period of time.
Onset of rainfall (being late by month)	Direct: if the onset of rainfall gets late in a given place, there will be shortage of moisture to grow crops following a regular crop calendar. Hence, the agroecosystem of a place with less variability of onset time of rainfall will be less sensitive to climate change. That is, there will be adequate moisture to propagate crops on time following a regular crop calendar.
Offset of rainfall (being earlier by month)	Direct: if the offset of rainfall gets earlier in a given place, there will be shortage of moisture to grow crops following a regular crop calendar. Hence, the agroecosystem of a place with less variability of offset time of rainfall will be less sensitive to climate change. That is, there will be adequate moisture to the final (flower and fruit) stage of a growing crop.
MATRF (CV in %)	Direct: if the variability (CV in %) of MATRF gets higher in a given place, there will be either shortage or excess water/moisture to grow crops. Hence, the agroecosystem of a place with less variability of MATRF will be less sensitive to climate change. That is, there will be optimum amount of moisture to grow crops following a regular crop calendar.
MMMaxT (CV in %)	Direct: if the variability (CV in %) of MMMaxT gets higher in a given place, there will be excess moisture loss through evapotranspiration to grow crops. Hence, the agroecosystem of a place with less variability of MMMaxT will be less sensitive to climate change. That is, there will be optimum moisture loss through evapotranspiration.
MMMinT (CV in %)	Direct: if the variability (CV in %) of MMMinT gets higher in a given place, there will be excess moisture loss through evapotranspiration to grow crops. Hence, the agroecosystem of a place with less variability of MMMaxT will be less sensitive to climate change. That is, there will be optimum moisture loss through evapotranspiration.
The coverage of arable land (LCC II & III %)	Indirect ∗: if the coverage of arable land gets higher in a given place, there will be more potential to grow crops. Hence, the agroecosystem of a place with more proportion of arable land will be less sensitive to climate change. That is, there will be adequate capacity to produce enough number of crops.
Crop diversity (count)	Indirect∗: if the crop diversity gets higher in a given place, there will be more capacity to absorb unprecedented climate related shocks. Hence, the agroecosystem of a place with more crop diversity will be less sensitive to climate change. That is, there will be a chance to manage possible crop shocks through using crop diversity as an insurance mechanism.

LGP = length of growing period; MATRF = mean annual total rainfall; MMMaxT = mean monthly maximum temperature; MMMinT = mean monthly minimum temperature; LCC = land capability class; CV = coefficient of variation; ∗ Inverted value computed.

As presented in the previous studies [37, 57], various indicators of agro–ecosystem sensitivity to climate change could be measured at different unit of measurement. Accordingly, it was essential to standardize the observed/estimated values (indices) employing a mathematical function. The need for standardizing values is to convert the indicators to relative rather than absolute values. The mathematical notation of Human Development Index–HDI (Eq. (2)) that could be used to standardize various indicators of livelihood profiles is developed by United Nations Development Program [68]. Then after, the same method was employed in various studies [37, 57]. For some of the indicators such as MATRF, MMMaxT, and MMMinT, it was found useful to compute variability among ACZs employing CV (%), Eq. (3).

(2) $$IndexV_{ACZ}=\frac{I_{A}-I_{W\min}}{I_{W\max}-I_{W\min}}$$

**Index**_**IA**_ = indexed value of an indicator in an agro–climatic zone; **I**_**A**_ = actual value of an indicator in an agro–climatic zone; **I**_**Wmin**_ = the actual minimum value of the same indicator over the watershed; and **I**_**Wmax**_ = the actual maximum value of the same indicator over the watershed.

(3) Coefficient of Variation(CV%)=(Standard Deviation/Mean)∗100

(4) Standard Error(SE)=Standard Deviation/Square Root of Sample Size

## 3 Results and discussion

### 3.1 Climate variability

The MARF for the entire Gilgel Abay watershed ranged from 1385 mm–2160 mm; whereas the MMMaxT and MMMinT were ~5°C–10 °C and ~21°C–27 °C, respectively. The value declines towards the higher elevation, Sub–Alpine. As per the computed value of CV (%), the inter–annual variability of the MARF (mm), MMMaxT (°C), and MMMinT (°C) were found to be different in the ACZs of the watershed. The CV (%) value showed the presence of variations of 7.85–11.21 (%), 7.21–10.34 (%), 16.37–39.61 (%) for MARF (mm), MMMaxT (°C), and MMMinT (°C), respectively across the ACZs of the watershed (Table 3). The inter-annual variability of both onset and offset time of rainy season was found to be in the range of 0.3–1.25 months. In all of the indicators of exposure to climate variability, the lowest and highest values were observed in the Moist–Cool and Sub–Alpine ACZs, respectively. The presence of climate variability in the country was also indicated in the previous studies [18, 27, 28, 29]. The inter–annual variability of Ethiopia's rainy season (June–September) was primarily governed by the El Niño/Southern Oscillation (ENSO) and secondarily reinforced by more local climate indicators near Africa and the Atlantic and Indian oceans [10].

**Table 3:** Analysis of agro–ecosystem sensitivity to climate variability in the Gilgel Abay watershed, Ethiopia.

Indicators	Moist–Cool			Cold			Moist–Cold			Sub–Alpine		
	mean	SE	(CV %)	mean	SE	(CV %)	mean	SE	(CV %)	mean	SE	(CV %)
MARF (mm)	1890	22.14	7.4	1575	22.93	9.2	1387	23.72	10.8	1385	23.88	10.9
MMMaxT (°C)	26.7	0.335	7.5	25.5	0.337	7.9	21.9	0.340	9.3	20.7	0.340	9.9
MMMinT (°C)	9.77	0.283	17.4	9.67	0.293	18.2	7.99	0.298	22.4	4.62	0.302	39.2

SE = standard error, CV = coefficient of variation, MARF = mean annual rainfall, MMMaxT = mean monthly maximum temperature, MMMinT = mean monthly minimum temperature.

Congruent to the result of the current study, previous studies [18, 37] showed that the presence of climatically diverse regions in the Ethiopian highlands could be considered as a challenge for policy–relevant implementation mainly because climatic and biophysical conditions vary dramatically within in short distances. Similarly, as per the result of [18], the level of climate variability and drought was found to be different in the agro–climatic zones of the Upper Blue Nile basin, Ethiopia. Similarly, as per the result of previous studies [47, 48, 59] climate change is predicted to have severe impacts on mountainous regions.

### 3.2 Land capability class

The elevation (m a.s.l) and slope of the Gilgel Abay watershed varies from 1786–3503 m and 0–45%, respectively. Over all, the steepness of the slope increases as one goes from Moist–Cool to Sub–Alpine direction of the study site. The result revealed that heavy clay, clay, and clay loam were observed to be the major texture classes in the Moist–Cool, Cold, Moist–Cold and Sub–Alpine ACZ zones, respectively. In terms of area coverage, 57.6%, 34.2 and 8.2 of the study watershed was found to be covered with clay, heavy clay and clay loam texture classes. The result confirms the existence of a spatial autocorrelation between soil texture and terrain for reasons related to balance of processes such as precipitation, infiltration, and runoff. The observed spatial distribution of soil texture in the Gilgel Abay infers the presence of low water holding capacity in the study watershed. The result also implies the existence of relatively severe erosion in the upstream and siltation in the downstream part of the Gilgel Abay watershed.

Overlaying soil texture class, slope and elevation zones, a LCC layer was generated. As per the result of the current study, the Gilgel Abay watershed comprises LCCs that varies from LCC II to LCC V (Table 4). LCC–III was found to be the most dominant in the study site, while LCC–V was situated only in the higher elevation zones: Sub–Alpine and Moist–Cold situated ACZs. In terms of the observed limitation of land, both arable and non–arable land were observed with a range of LCCII– LCC V. The proportion of arable land (LCC II and III) varied from 13.30% (in the Sub–Alpine) to 93.00% (in the Moist–Cold). In congruent with the result of the current study, the study of [40, 69] that were conducted in the Ethiopian highlands indicated the presence of LCC that ranges from class II to V. Similarly, the study of [61] conducted in the Ethiopian highlands, showed the presence of LCC that ranges from LCC I to LCC IV.

**Table 4:** A spatial coverage of LCCs in the ACZs of Gilgel Abay watershed, Ethiopia.

LCC	Area Coverage (%) the ACZs			
	Moist–Cold	Cold	Moist–Cool	Sub–Alpine
II	51.7	2.2	1.5	1.0
III	40.8	57.1	15.0	12.8
IV	7.4	40.6	43.7	50.6
V	0.1	0.1	39.8	35.6

LCCs = land capability classes; ACZs = agro–climatic zones.

Likewise, previous studies [37, 40, 42] showed similar results in that the degree of land limitation is larger in the upstream portion of the watershed possibility for reasons related to the nature of topography and level of soil erosion.

### 3.3 Crop diversity and length of crop growing period

Barley *(Hordeum Vulgare)*, potatoes (*Solanum Tuberosum*), wheat (*Triticum Spp*), onion (*Allium cepa*), horse bean (*Vicia Faba*), pea (*Pisum* Sativum), *tef* (*Eragrostis)*, finger millet (*Eleusine Coracana)*, maize (*Zea Mays)*, and Niger seed *(Guizotia abyssinica)* are the major field crops in the Gilgel Abay watershed. Previous studies [36, 37] also showed similar results. In terms of mean annual rainfall, all of the ACZs received sufficient rainfall (1228–1640 mm) to grow the local food crops; i.e., cereals and pulses [36]. Yet, apart from ATRF, difference in elevation zone, terrain, soil property, and temperature are important in determining LGP in a given geographical zone. As temperature gets cooler, the LGP will be longer [36]. As per the result, the current LGP (month) was found to be in the range of 3.25–6.25; whereas crop diversity (count) from 2 to 7 in the ACZs of the Gilgel Abay watershed (Table 5). The LGP increases towards the Sub–Alpine ACZ; whereas, crop diversity gets lower towards the same ACZ. The production of red onion (allium cepa), oat (*Avena sativa*), local wheat (*Triticum*), and pea (*Pisum sativum*) was abandoned in the upstream. Similarly, the production of linseed (*Linmu usitatisimum*), barley (*Hordeum vulgare*), and niger *(Guizotia abyssinica)* was abandoned in the downstream. Yet, starting from the last decade, crops like maize and tef became the common crops in high elevation areas, Cold ACZ, possibly because of global warming. Future studies could focus on potential and specific causes for the observed change in the spatial distribution of crops.

**Table 5:** The current average crop diversity (Dty) in count and LGP (month) in the ACZs of Gilgel Abay watershed, Ethiopia.

	Moist–Cool			Cold			Moist–Cold			Sub–Alpine		
		Dty	LGP		Dty	LGP		Dty	LGP		Dty	LGP
Crops	tef, maize, finger millet, wheat, onion, potato and niger seed	7	3.25	barley, wheat, potato, pea, horse bean, onion, tef and maize	7	4.25	barley, wheat, potato, pea and horse bean	5	5.25	barley, wheat and potato	3	6.25

LGP = length of growing period, ACZ = agro–climatic zones; Barley *(Hordeum Vulgare)*, potatoes (*Solanum Tuberosum*), wheat (*Triticum* *Spp*), onion (*Allium cepa*), horse bean (*Vicia Faba*), pea (*Pisum* Sativum), *tef* (*Eragrostis)*, finger millet (*Eleusine Coracana)*, maize (*Zea Mays)*, and Niger seed *(Guizotia abyssinica).*

**Figure 5:**
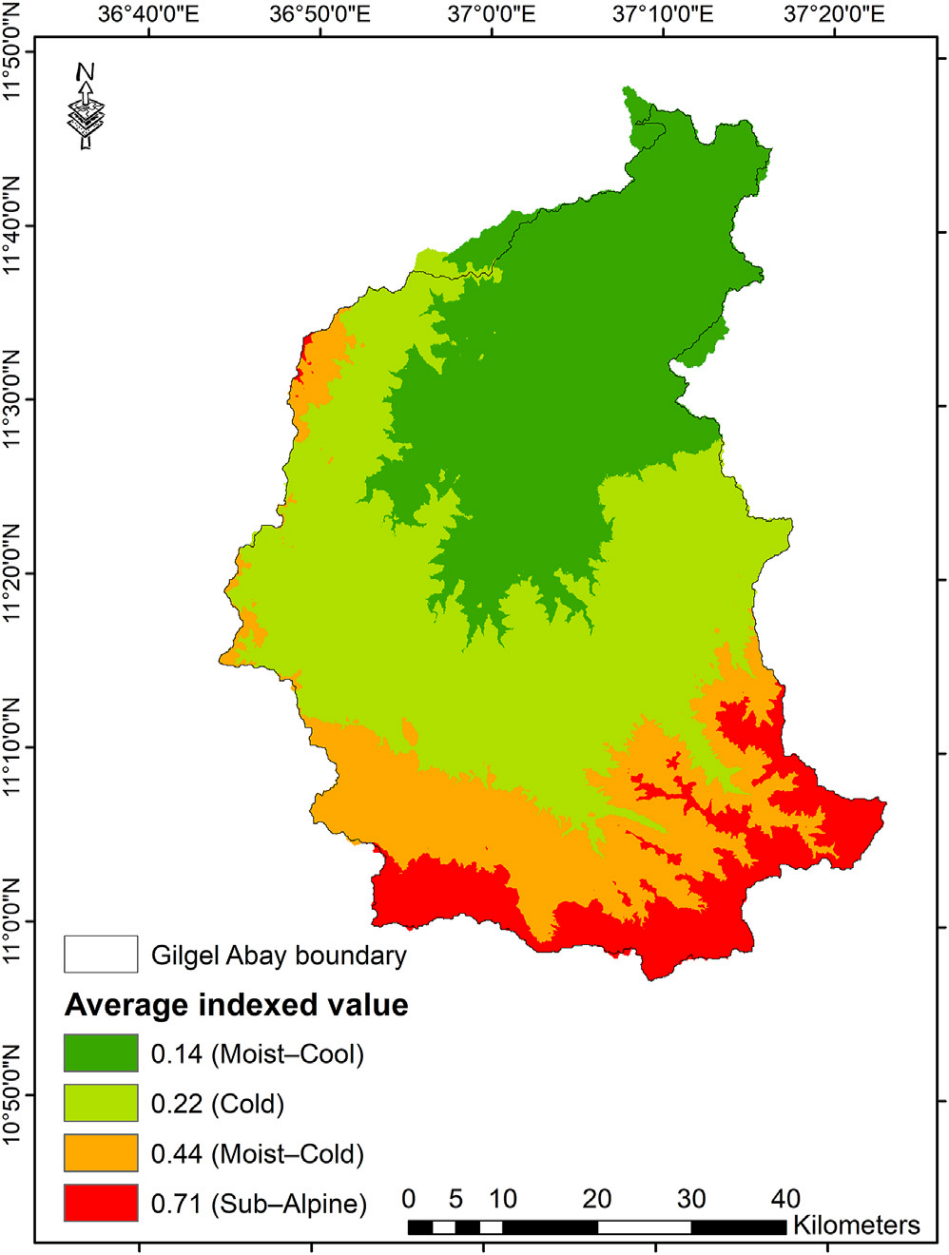
The degree of agro–ecosystem sensitivity to climate change among the agro–climatic zones of Gilgel Abay Watershed, Ethiopia.

Finally, to compare the level of agro–ecosystem sensitivity to climate change among the ACZs of Gilgel Abay watershed, the observed values of LCC (%), minimum and maximum temperature (°C), rainfall (mm), LGP (month) and crop diversity (count) were indexed (standardized) and aggregated (Table 6 and Figure 5). The indexed value ranged from 0.14–0.71 over the Gilgel Abay watershed. The highest and lowest indexed values were observed in the *Sub–Alpine* and Moist–Cool ACZs, respectively.

**Table 6:** The indexed values of agro–ecosystem sensitivity to climate change among the ACZs of Gilgel Abay watershed, Ethiopia.

Indexed values for ACZs										
Indicators	Watershed (max)	Watershed (min)	Moist–Cool (average)	Index	Cold (average)	Index	Moist–Cold (average)	Index	Sub–Alpine (average)	Index
LGP (month)	6.50	3.00	3.50	0.14	4.30	0.37	5.30	0.66	6.30	0.94
Onset of rainfall (being late by month)	1.25	0.30	0.48	0.19	0.48	0.19	0.72	0.44	1.10	0.84
Offset of rainfall (being earlier by month)	1.25	0.30	0.48	0.19	0.48	0.19	0.73	0.45	1.10	0.84
MATRF (CV in %)	11.21	7.85	8.78	0.28	9.61	0.52	10.92	0.91	10.99	0.93
MMMaxT (CV in %)	10.34	7.21	7.65	0.14	8.05	0.27	8.91	0.54	9.80	0.83
MMMinT (CV in %)	39.61	16.37	16.65	0.01	17.95	0.07	23.94	0.33	38.64	0.96
Arable land (LCC II & III %)∗	93.00	13.30	92.50	0.01	59.30	0.02	16.50	0.06	13.50	0.07
Crop diversity (count)∗	7.00	2.00	6.50	0.13	7.00	0.13	5.00	0.17	3.00	0.25
Indexed value (average)				0.14		0.22		0.44		0.71

ACZ = agro–climatic zones; max = maximum; min = minimum; MARF = mean annual rainfall; MMMaxT = mean daily max temperature; MMMinT = mean daily min temperature; LCC = land capability class; ∗ inverted value computed.

Overall, by considering the variables analyzed in the current study, the degree of agro–ecosystem sensitivity to climate change was found to be higher towards the Sub–Alpine ACZ of the Gilgel Abay watershed. Consistent to the current study, the result of [18, 37] reveal that the extreme highland agro–climatic zones (Sub–Alpine and Moist–Cold) have the highest perceived vulnerable zone to climate stresses. Similarly, the presence of local variation in resilience to climate change was also indicated in the previous studies [47, 48, 66]. As per the result of a case study, in relative terms, communities in the high–altitude zones were more vulnerable probably due to high exposure to extreme events that can affect agricultural production negatively [70]. Compared to the plain area, more proportion of smallholder farmers in the hilly zone were highly sensitive to climate change [71].

## 4 Conclusions and recommendations

Employing advanced technology together with local peoples’ experience was found to be useful in the study of a comprehensive agro–ecosystem sensitivity to climate change in the Gilgel Abay watershed which comprises arable and non–arable land segments. The degree of land limitation, inter–annual variability of climate (MARF mm, MMMinT° C, and MMMaxT, °C), variability in onset and offset time of rainy season, and the actual LGP increased towards the Sup–Alpine ACZ. On the other hand, the diversity of the actual local food crops increased towards the Moist–Cool ACZ. Yet, starting from the last decade, crops like maize and tef became common crops in the high elevation part of the watershed, Cold ACZ, possibly because of temperature increment. In relative terms, the degree of agro–ecosystem sensitivity to climate change was higher towards the Sub–Alpine ACZ.

In order to minimize the degree of agro–ecosystem sensitivity to climate change, conservation agriculture and land capability–based land use, and application of early maturing crops need be enforced more in the Sub–Alpine and Moist–Cold ACZs of the watershed. Overall, in order to minimize the degree of rural livelihood vulnerability to climate change, the type of local development interventions to be made in the various ACZs need to be determined/prioritized considering the level of agro–ecosystem sensitivity to climate change.

## Declarations

### Author contribution statement

Mintesinot A. Taye: Conceived and designed the experiments; Performed the experiments; Analyzed and interpreted the data; Contributed reagents, materials, analysis tools or data; Wrote the paper.

### Funding statement

This work was supported by 10.13039/501100005872Bahir Dar University, Ethiopia.

### Data availability statement

Data will be made available on request.

### Declaration of interests statement

The authors declare no conflict of interest.

### Additional information

No additional information is available for this paper.
